# Association of clinical and laboratory variables with in-hospital incidence of deep vein thrombosis in patients after acute ischemic stroke: A retrospective study

**DOI:** 10.1097/MD.0000000000024601

**Published:** 2021-02-12

**Authors:** Yucai Huang, Cuirong Guo, Kun Song, Changluo Li, Ning Ding

**Affiliations:** Department of Emergency Medicine, Changsha Central Hospital, University of South China.

**Keywords:** acute ischemic stroke, D-dimer, deep vein thrombosis, muscle tone, pulmonary infection

## Abstract

Deep vein thrombosis (DVT) is a serious complication in patients with acute ischemic stroke (AIS). Early prediction of DVT could enable physicians to perform a proper prevention strategy. We analyzed the association of clinical and laboratory variables with DVT to evaluate the risk of DVT in patients after AIS.

AIS patients admitted to the Changsha Central Hospital between January 2017 and December 2019 with length of stay in hospital ≥7 days were included. Clinical and laboratory variables for DVT at baseline were collected, and the diagnosis of DVT was confirmed by ultrasonography. Independent factors were developed by Multivariate logistic regression analysis.

A total of 101 patients were included in the study. The in-hospital incidence of DVT after AIS was 19.8%(20/101). The average level of D-dimer when DVT detected was significant increased around 4-fold than that on admission (*P* < .001). Pulmonary infection (odds ratio [OR] = 5.4, 95%CI:1.10–26.65, *P* = .037)) and increased muscle tone (OR = 0.11, 95%CI:0.02–0.58, *P* = .010) as independent relevant factors for DVT were confirmed.

Pulmonary infection as a risk factor and increased muscle tone as a protective factor for DVT were identified in patients after AIS. The level of D-dimer which increased around 4-fold compared to the initial level could be an indicator for DVT occurrence.

## 1 Introduction

Acute ischemic stroke (AIS) is 1 of the severe cerebral disorders leading to high disability and mortality among hospitalized patients,^[1]^ while deep vein thrombosis (DVT) is 1 of most common and fatal complications in patients with AIS.^[2]^ In western countries, the incidence of DVT in acute stroke patients without prophylactic treatment was up to around 80%,^[3]^ and DVT still occurred in at 2% to 3% of patients even receiving comprehensive prophylactic therapy.^[4,5]^ The CLOTS trial, as a largest multicenter observational research with 5632 patients with stroke revealed that the in-hospital incidences of detected DVT within 10 days and within 30 days were 11% and 15%, respectively.^[6]^ In Asia, the occurrence of DVT after stoke varied in 3% to 17%.^[7]^

However, the clinical outcomes were confirmed to be improved significantly in DVT patients after AIS including thrombolytic and anti-coagulation therapies,^[8]^ there was still lack of explicitness on the timing of thromboprophylaxis in the international guidelines.^[1]^ Hence, the risk of developing DVT in every single patient after AIS should be evaluated early so that the benefit and risk of thromboprophylaxis therapy could be comprehensively weighed and analyzed. In this study, we analyzed the association of clinical and laboratory variables with DVT in patients after AIS in order to explore some factors for predicting in-hospital incidence of DVT.

## 2 Methods

### 2.1 Patients

Patients with AIS admitted to the Changsha Central Hospital between January 2017 and December 2019 with length of stay in hospital≥7 d were included. Inclusion criteria were identified as follows: age ≥18, radiographical results showing cerebral infarction, and length of stay in-hospital ≥7 days. Exclusion criteria were identified as follows: past medical history of DVT, DVT detected on admission, varicose of lower extremities, malignant tumor, and coagulation disorders.

### 2.2 Data collection

Based on electronic health records, the general information of patients was collected, including age, sex and comorbidities (coronary heart disease, rheumatoid heart disease, hypertension, diabetes). The National Institutes of Health Stroke Scale, Glasgow Coma Scale, Wells scale and Modified Rankin Scale were performed for all the patients when on admission. Laboratory variables while patients admitted in ≤24 hours were collected including platelet counts, red blood cell volume distribution width, low density lipoprotein, D-dimer and fibrinogen. Moreover, management therapies including anti-coagulation and rehabilitation were also recorded. When DVT detected in patients by color doppler ultrasonography (CDUS), the clinical variables in 24 hours were collected. The incidence of in-hospital pulmonary infection and in-bed≥3 days were recorded. Clinical outcomes were length of stay in hospital, in-hospital incidence of pulmonary embolism and in-hospital mortality.

### 2.3 DVT and muscle tone assessment

According to the electronic health record, the DVT assessment was applied with CDUS on the patients while on admission. Common femoral vein and the popliteal vein of patients were examined by CDUS for DVT diagnosis. During the hospitalization, CDUS was performed in almost every one week after admission as well as whenever clinically requested such as swollen or paresthesia of extremities on the basis of electronic health records. Muscle tone was evaluated everyday by physicians based on modified Ashworth scale. According to modified Ashworth scale, there are 5 grades (0 to 4) in muscle tone. When the affected limbs were passively moved and no resistance occurred, it was grade 0 in muscle tone, which also was defined as normal muscle tone. When resistance occurred, it was defined as muscle tone increased. With the extent of resistance increasing, the grade of muscle tone also increased. The day when muscle tone increased also recorded during the hospitalization. All the medical information mentioned above was based on clinical medical records.

### 2.4 Statistics

Statistical results were showed in mean ± standard deviation for normal data, while for non-normal data, interquartile range and median were utilized. Categorical data were showed as percentage and number. The comparison between 2 groups was performed with chi-squared test or Mann–Whitney *U*-test. Variables that were significant different in 2 groups on univariate analysis were further analyzed in multivariate logistic regression. Statistical analysis was performed using SPSS software (version 26) and 2-sided *P* values of less than .05 were defined statistically significant.

## 3 Results

### 3.1 General characteristics of the patients

**Figure 1:**
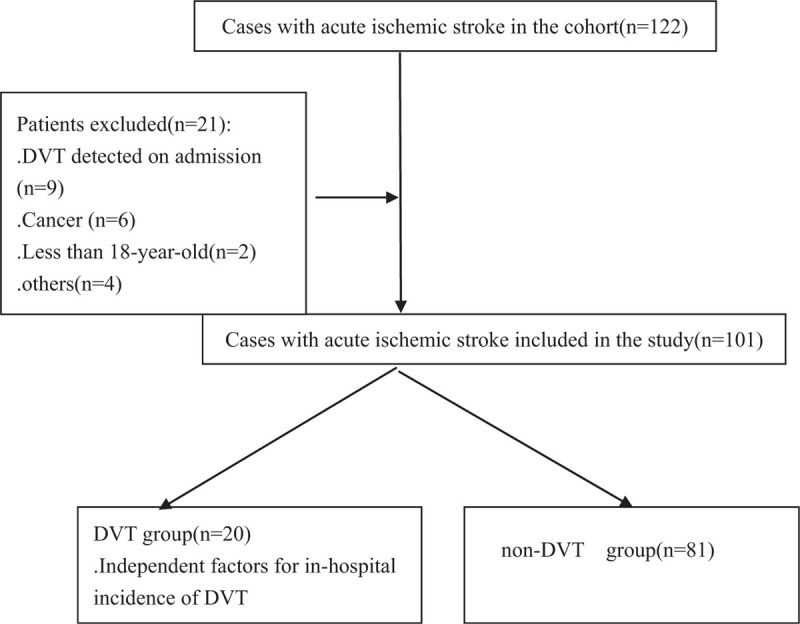
Flow chart for patients enrollment and study design.

A total of 122 patients with AIS were enrolled and 21 were excluded on the basis of exclusion criteria (Fig. 1). Finally, 101 patients were included in the study. 66% (67/101) were male and median age was 66 (66.0 ± 16.2). There were 20 patients in DVT group and 81 patients in non-DVT group, respectively. The general characteristics of the patients were demonstrated in Table 1. There were no significant difference in sex, age, comorbidities (coronary heart disease, rheumatoid heart disease, hypertension, diabetes) between 2 groups. Lab variables (platelet counts, red blood cell volume distribution width, low density lipoprotein, fibrinogen), management (anti-coagulation, rehabilitation therapy) and clinical outcomes were not significant different between 2 groups. None of the patients had in-hospital pulmonary embolism. In DVT group, the proportion of increased muscle tone was significant lower than that in non-DVT group (10% vs 67.8, *P* = .002), while there was no significant difference in muscle strength between 2 groups. The incidence of pulmonary infection was significant higher in DVT group than non-DVT group (85% vs 60.4%, *P* = .044). The level of D-dimer and Wells scale were also significant different (*P* < .05).

**Table 1:** Baseline characteristics of the patients (n = 101).

Characteristic	Total (n = 101)	DVT (n = 20)	Non-DVT (n = 81)	*P*-value
Gender				.292
Male, n (%)	67 (66.3)	11 (55.0)	56 (69.1)	
Female, n (%)	34 (33.7)	9 (45.0)	20 (30.9)	
Age (yr, mean ± SD)	66.0 ± 16.2	65 ± 16.4	66 ± 16.3	.684
Comorbidities				
Coronary heart disease, n (%)	23 (22.7)	8 (40.0)	15 (18.5)	.070
Rheumatoid heart disease, n (%)	6 (5.9)	1 (5.0)	5 (6.1)	1.000
Hypertension, n (%)	83 (82.1)	18 (90.0)	65 (80.2)	.514
Diabetes,n (%)	15 (14.8)	2 (10.0)	13 (16.0)	.729
In-hospital complications				
In-bed≥3d,n (%)	85 (84.1)	17 (85.0)	68 (83.9)	1.000
Pulmonary infection, n (%)	66 (65.3)	17 (85.0)	49 (60.4)	.044
Central venous catheter, n (%)	6 (5.9)	2 (10.0)	4 (4.9)	.340
Increased muscle tone n (%)	40 (39.6)	2 (10.0)	38 (67.8)	.002
Muscle strength (grade, mean ± SD)	2.4 ± 1.6	2.8 ± 1.7	2.4 ± 1.6	.332
Lab findings				
Platelet (10^9/L, mean ± SD)	189.3 ± 57.7	182 ± 50.5	191 ± 59.5	.512
RDW (%, mean ± SD)	14.6 ± 12.3	13.7 ± 2.1	14.9 ± 1.5	.704
LDL (mmol/L, mean ± SD)	2.0 ± 1.2	1.9 ± 1.2	2.0 ± 1.1	.813
D-dimer (mg/L, mean ± SD)	1.6 ± 3.3	3.1 ± 5.3	1.3 ± 2.4	.021
Fbg (mg/L, mean ± SD)	2.9 ± 1.3	2.5 ± 1.1	3.0 ± 1.3	.068
Scoring system				
GCS	11.6 ± 3.5	11.5 ± 3.5	11.6 ± 3.5	.927
NIHSS	16.9 ± 10.2	19.7 ± 10.6	16.2 ± 10.1	.181
MRS	4.1 ± 1.3	4.2 ± 1.4	4.1 ± 1.3	.905
Wells	0.43 ± 0.64	0.7 ± 0.7	0.4 ± 0.6	.031
Management				
Anti-coagulation n (%)	8 (7.9)	1 (5.0)	7 (8.6)	1.000
Rehabilitation therapy n (%)	101 (100.0)	20 (100.0)	81 (100.0)	1.000
Clinical outcomes				
Pulmonary embolism n (%)	0 (0.0)	0 (0.0)	0 (0.0)	1.000
Length of stay in hospital (days)	79.0 ± 60.2	73.7 ± 37.6	80.3 ± 64.7	.666
In-hospital mortality, n (%)	1 (0.9)	0 (0.0)	1 (1.2)	1.000

DVT = deep vein thrombosis, Fbg = fibrinogen, GCS = Glascow coma scale, LDL = low density lipoprotein, MRS = modified Rankin scale, NIHSS = National Institutes of Health Stroke Scale, RDW = red blood cell volume distribution width, SD = standard deviation.

### 3.2 Multiple logistic regression analysis for in-hospital incidence of DVT

Two independent variables were identified by multivariate logistic regression analysis in (Table 2). Pulmonary infection was a risk factor for in-hospital incidence of DVT (odds ratio [OR] = 5.4, 95%CI:1.10–26.65, *P* = .037)), while increased muscle tone was negative parallel with in-hospital incidence of DVT (OR = 0.11, 95%CI:0.02–0.58, *P* = .010).

**Table 2:** Multiple logistic regression analysis for in-hospital incidence of DVT.

	OR	95%CI	*P*-value
Coronary heart disease	2.4	0.48–12.24	.288
Atrial fibrillation	2.5	0.49–13.2	.265
Fbg	0.6	0.34–1.13	.116
D-dimer	1.2	0.91–1.47	.231
Increased muscle tone	0.11	0.02–0.58	.010
Wells score	2.0	0.86–4.62	.106
Pulmonary infection	5.4	1.10–26.65	.037

CI = confidence interval, DVT = deep vein thrombosis, Fbg = fibrinogen.

### 3.3 Analysis of the relevance between DVT and increased muscle tone

In non-DVT group, the incidence of increased muscle tone was significant higher than that in DVT group **(**67.8% [n = 38] vs10% [n = 2**]**, *P* = .002). Non-parametric correlation analysis showed that there was a negative correlation between DVT and increased muscle tone (R = -0.703, *P* = .031). Compared the time when DVT detected and when muscle tone increased, the average days from admission to when DVT detected were significantly longer than the time from admission to when muscle tone increased (17.7 vs 9.9, *P* < .001).

### 3.4 Analysis of the changes in laboratory variables when DVT detected

Compared the levels of laboratory variables in patients with DVT between the time on admission and the time when DVT detected, the average level of D-dimer when DVT detected was significant increased around 4-fold than that on admission (13.6 ± 1.7vs 3.1 ± 5.3, *P* < .001)(Table 3).

**Table 3:** Comparison laboratory variables in different time.

	At admission	At Time when DVT detected	*P*-value
Platelet (10^9^/L, mean ± SD)	182 ± 50.5	172 ± 30.5	.483
RDW (%, mean ± SD)	13.7 ± 2.1	13.9 ± 4.9	.658
LDL (mmol/L, mean ± SD)	1.9 ± 1.2	1.8 ± 1.3	.337
D-dimer (mg/L, mean ± SD)	3.1 ± 5.3	13.6 ± 1.7	<.001
Fbg (mg/L, mean ± SD)	2.5 ± 1.1	2.8 ± 0.9	.198

DVT = Deep Vein Thrombosis, Fbg = fibrinogen, LDL = low density lipoprotein, RDW = red blood cell volume distribution width, SD = standard deviation.

## 4 Discussion

Risk factors for DVT in patients after acute stroke varied in different clinical researches. The typical factors included older age, medical history of DVT, increased body mass index, malignant tumor, pulmonary infection, increased level of some laboratory variables.^[9–13]^ In our study, pulmonary infection and increased muscle tone were identified as independent factors associated with in-hospital incidence of DVT in patients after AIS.

In our multiple logistic regression model, patients with pulmonary infection experienced an increased risk of DVT. A higher risky relevance of pulmonary infection with DVT was also demonstrated in other researches.^[14,15]^ A research on the psychiatric inpatients revealed that the average in-hospital incidence of DVT was up to 10%, while the DVT risk in the group with pulmonary infection was significantly increased.^[16]^ In addition, a clinical case review showed that patients died secondary to staphylococcal community-acquired pneumonia had higher risk of DVT.^[14]^ Research clarified that some pathogens, especially bacteria had surface proteins and exotoxins leading to damaging endothelial cells, activating coagulation pathway and forming micro-thrombosis and DVT.^[17]^ A meta-analysis including 3551 stroke patients showed that intermittent pneumatic compression (IPC) significantly reduced the risk of DVT,^[18]^ which could be partly explained by that IPC was an effective management for ameliorating pulmonary infection.

Immobility was a major risk factor for DVT in neurological diseases.^[4,19]^ An observational research analyzed 542 stroke patients with DVT and found that DVT occurred in 73% of patients with weaker muscle strength while only 11% of patients with stronger were diagnosed with DVT.^[20]^ Our study showed that there was no difference in muscle strength between DVT-group and non-DVT group. Although muscle strength was not linked with DVT in our study, muscle tone was identified as a negative relevant factor with the incidence of DVT and patients with increased muscle tone were less likely to developing DVT. Among patients with stroke, increased muscle tone and muscle spasms of lower extremities usually develop gradually within several months,^[20]^ which theoretically resulted in emptying of veins in lower extremities by enhancing the capability of the calf muscle pump. Previous studies observed some vascular changes with a generalized atrophy of the arteries and decreased blood flow in the paralyzed lower extremities, which could adjust the lower oxygen supply to match the decreased activity of the paralyzed muscles.^[21]^ With blood stasis reduced in extremities, the risk of DVT was decreased. Moreover, clinical observations suggested that increased muscle tone was a protective factor against DVT in neurological disorders.^[22,23]^ Increased muscle tone in stroke patients at the initial stage indicated the gradual emergence of active exercise, which could lead to increase the cerebral blood flow in the injured site and promote the recovery of motor function and intelligence, resulting in blood flow velocity of hemiplegic extremities increased and the occurrence of DVT decreased.^[24,25]^

Interestingly, we compared the laboratory variables in different times and found that the level of D-dimer was significantly higher when DVT detected than that on admission, which suggested that dynamically testing D-dimer could be a predictive method for DVT. D-dimer as a sensitive marker for thrombus formation, was an indicator for predicting DVT in different disorders.^[12,15,26]^ A recent prospective observation study with 452 stroke patients concluded that with the median of D-dimer (0.38 FEU mg/L) as cutoff value, patients with higher level of D-dimer had a higher risk of DVT.^[27]^ D-dimer demonstrated a sensitivity of 85% to 95% and a specificity of 25% to 50% for DVT.^[28,29]^ Baseline levels of D-dimer varied in different age due to variability in the inflammatory and immune response dependent on age^[30]^ and the elders were more likely to suffering from stroke, which could explain why the specificity of a standard D-dimer cut-off at 500ug/L for DVT prediction in elderly patients with stroke was comparatively low. A systemic review indicated that utility of an age-adjusted D-dimer cut-off (patient's age∗10) ug/L) for elderly patients for ruling out DVT was recommended.^[31]^ The average levels of D-dimer when DVT detected were about 4-fold as the levels of that on admission in our study, which suggested that the gradually increased level of D-dimer was associated with DVT. However, D-dimers are not very specific, they can also be elevated in some disorders without DVT.^[32]^ A recent research in Korea concluded that female sex and a high National Institutes of Health Stroke Scale score were independently associated with the risk of DVT, while D-dimer level was not,^[33]^ which differed from our study maybe due to the different cohorts in different countries.

The strength of this study is that it concludes that pulmonary infection as a risk factor and increased muscle tone as a protective factor for DVT, which enables physicians to take early managements to reduce the incidence of DVT such as paying more attention to the patients with pulmonary infection and taking more effective therapies to improve muscle tone of patients. Early managements for preventing pulmonary infection such as IPC or some medicines for reducing sputum secretion should be applied in patents with AIS. Effective and comprehensive rehabilitation in early stage also played an important role in improving muscle tone and reducing the risk of DVT. In addition, the significant change in level of D-dimer could be a warning for DVT occurrence so that dynamically monitoring the level of D-dimer is of importance in patients after ischemic stroke. Our study showed that the level of D-dimer which increased around 4-fold compared to the initial level could be an indicator for DVT occurrence.

Limitations also should be clarified. First, our study enrolled relatively small samples and further study with larger samples and more subtypes of stroke needs to be explored. Second, because it was a retrospective research, the time when DVT detected by CDUS might be delayed compared to the actual time when DVT developed. Further prospective research should be performed to validate our conclusions. Moreover, Caution should be needed while our findings is interpreted in other multiple-center cohort studies. Third, although venography was the gold standard for diagnosing DVT, serial compression ultrasonography as the recommended test was applied in our study for detecting DVT owing to its non-invasiveness. It might be not as perfectly accurate as venography. Fourth, this study was performed based on medical records. Due to some data missing, not all the variables including use of oral contraceptives, history of surgery, fractures, and use of chemotherapy drugs in the past were assessed. Further research with larger samples and more variables should be explored for comprehensively analyzing the association of different factors with DVT as well as clinical characteristics and outcomes. Moreover, in the future, researches should pay more attention to the management including pharmacological thromboprophylaxis and rehabilitation in the early-stage of AIS.

## 5 Conclusion

Pulmonary infection as a risk factor and increased muscle tone as a protective factor for DVT were identified in patients after acute ischemic stroke. The level of D-dimer which increased around 4-fold compared to the initial level could be an indicator for DVT occurrence.

## Author contributions

The manuscript writing and patient's data recording were done by Yucai Huang and Ning Ding. Cuirong Guo and Kun Song assisted in information collection. Changluo Li and Ning Ding analyzed and interpreted the patients’ general indices. The final manuscript was read and ratified by all authors.

**Conceptualization:** Yucai Huang, Changluo Li.

**Data curation:** Cuirong Guo, Kun Song.

**Investigation:** Cuirong Guo, Kun Song.

**Writing – original draft:** Yucai Huang, Changluo Li.

**Writing – review & editing:** Ning Ding.

## Abbreviations

Abbreviations: AIS = acute ischemic stroke, CDUS = color doppler ultrasonography, DVT = deep vein thrombosis, IPC = intermittent pneumatic compression, OR = odds ratio.
